# Exploring clinical markers of Axon degeneration processes in Chemotherapy-induced peripheral neuropathy among young adults receiving vincristine or paclitaxel

**DOI:** 10.1186/s12883-024-03877-9

**Published:** 2024-09-28

**Authors:** Robert Knoerl, Emanuele Mazzola, Maria Pazyra-Murphy, Birgitta Ryback, A. Lindsay Frazier, Roy L. Freeman, Marilyn Hammer, Ann LaCasce, Jennifer Ligibel, Marlise R. Luskin, Donna L. Berry, Rosalind A. Segal

**Affiliations:** 1 Phyllis F. Cantor Center for Research in Nursing and Patient Care Services, Dana-Farber Cancer Institute, Boston, MA USA; 2 Present Address: University of Michigan School of Nursing, 400 North Ingalls St, Office 2350, Ann Arbor, MI 48109 USA; 3 Department of Biostatistics and Computational Biology, Dana-Farber Cancer Institute, Boston, MA USA; 4 Department of Cancer Biology, Dana-Farber Cancer Institute, Boston, MA USA; 5 Metabolomics Core, Dana-Farber Cancer Institute, Boston, MA USA; 6 Department of Pediatric Oncology, Dana-Farber Cancer Institute, Boston, MA USA; 7 Department of Neurology, Beth Israel Deaconess Medical Center, Boston, MA USA; 8 Department of Medical Oncology, Dana-Farber Cancer Institute, Boston, MA USA; 9 Biobehavioral Nursing and Health Informatics, University of Washington, Seattle, WA USA; 10 Deparment of Neurobiology, Harvard Medical School, Boston, MA USA

**Keywords:** Chemotherapy-induced peripheral neuropathy, NAD, human [Supplementary Concept], Young Adult, Neoplasms

## Abstract

**Background:**

Approximately 70% of patients receiving neurotoxic chemotherapy (e.g., paclitaxel or vincristine) will develop chemotherapy-induced peripheral neuropathy. Despite the known negative effects of CIPN on physical functioning and chemotherapy dosing, little is known about how to prevent CIPN. The development of efficacious CIPN prevention interventions is hindered by the lack of knowledge surrounding CIPN mechanisms. Nicotinamide adenine dinucleotide (NAD^+^) and cyclic-adenosine diphosphate ribose (cADPR) are potential markers of axon degeneration following neurotoxic chemotherapy, however, such markers have been exclusively measured in preclinical models of chemotherapy-induced peripheral neuropathy (CIPN). The overall objective of this longitudinal, observational study was to determine the association between plasma NAD^+^, cADPR, and ADPR with CIPN severity in young adults receiving vincristine or paclitaxel.

**Methods:**

Young adults (18–39 years old) beginning vincristine or paclitaxel were recruited from Dana-Farber Cancer Institute. Young adults completed the QLQ-CIPN20 sensory and motor subscales and provided a blood sample prior to starting chemotherapy (T1) and at increasing cumulative vincristine (T2: 3–5 mg, T3: 7–9 mg) and paclitaxel (T2: 300–500 mg/m^2^, T3: 700–900 mg/m^2^) dosages. NAD^+^, cADPR, and ADPR were quantified from plasma using mass spectrometry. Metabolite levels and QLQ-CIPN20 scores over time were compared using mixed-effects linear regression models and/or paired two-sample tests.

**Results:**

Participants (*N* = 50) were mainly female (88%), white (80%), and receiving paclitaxel (78%). Sensory and motor CIPN severity increased from T1–T3 (*p* < 0.001). NAD^+^ (*p* = 0.28), cADPR (*p* = 0.62), and ADPR (*p* = 0.005) values decreased, while cADPR/NAD^+^ ratio increased from T1–T3 (*p* = 0.50). There were no statistically significant associations between NAD + and QLQ-CIPN20 scores over time.

**Conclusions:**

To our knowledge, this is the first study to measure plasma NAD^+^, cADPR, and ADPR among patients receiving neurotoxic chemotherapy. Although, no meaningful changes in NAD^+^, cADPR, or cADPR/NAD^+^ were observed among young adults receiving paclitaxel or vincristine. Future research in an adequately powered sample is needed to explore the clinical utility of biomarkers of axon degeneration among patients receiving neurotoxic chemotherapy to guide mechanistic research and novel CIPN treatments.

**Supplementary Information:**

The online version contains supplementary material available at 10.1186/s12883-024-03877-9.

## Introduction

The symptoms of chemotherapy-induced peripheral neuropathy (CIPN) following neurotoxic chemotherapy administration (e.g., vincristine or paclitaxel) include bilateral, upper/lower extremity sensory (e.g., numbness, tingling, pain) and/or motor impairments (e.g., muscular weakness) [1] that may negatively affect physical function [2–4]. Resultantly, CIPN-induced reductions in physical function may necessitate chemotherapy dose modifications, thereby increasing mortality risk because patients are not receiving the optimal chemotherapy dose. Despite the known negative effects of CIPN, there are no recommended treatments for CIPN prevention [5]. The development of efficacious CIPN prevention interventions is hindered by the lack of knowledge surrounding CIPN mechanisms [6]. The first step towards designing effective CIPN prevention clinical trials is to gain a greater understanding of CIPN mechanisms.

Chemotherapy-induced peripheral neuropathy is characterized by a dying back axon degeneration [7], in which the most distal nerve endings are affected and intraepidermal nerve fiber innervation decreases [8]. Vincristine and paclitaxel, microtubule-targeting chemotherapy agents, are thought to induce axon degeneration by interfering with anterograde and/or retrograde axonal transport of proteins, organelles, and mRNAs [8–10]. Deficits in axonal transport of nicotinamide nucleotide adenylyltransferase 2 (NMNAT2) are thought to lead to the activation of sterile-α and Toll/interleukin 1 receptor motif containing protein 1 (SARM1), which is the main executioner of axon degeneration [10, 11]. Other potential contributors to vincristine or paclitaxel-induced axon degeneration include mitochondrial toxicity and alterations in intracellular calcium homeostasis [7].

One potential therapeutic target of axon degeneration is nicotinamide adenine dinucleotide (NAD^+^) [12], a cofactor that plays important roles in cell metabolism and signaling [13]. NAD^+^ decreases following axonal injury in part due to loss in active NMNAT2 function, which synthesizes NAD from nicotinamide mononucleotide [NMN] [10]. Recent evidence demonstrates that an increase in NMN/NAD^+^ ratio following NMNAT2 loss activates SARM1 [14]. Subsequently, SARM1 activation further accelerates NAD^+^ loss and breaks down NAD^+^ to cyclic-adenosine diphosphate ribose (cADPR), or ADPR and nicotinamide [11, 15, 16]. Increased SARM1-mediated cyclic ADPR production leads to an influx of calcium in the axons and accelerates SARM1 dependent axon degeneration, while loss of NAD + results in a lower energetic state [17]. Currently, the ratio of cADPR/NAD^+^ is thought to be a promising marker of relative SARM1 activity as the ratio accounts for SARM1 enzymatic activity (i.e., SARM1 breaks down NAD^+^ into cADPR) and substrate depletion (i.e., NAD^+^ rapidly decreases in response to SARM1 activity) [14, 18].

While NAD^+^ and cADPR are potential markers of SARM1 activity [19], to date, such markers have been exclusively measured in nerves [18, 19]. To our knowledge, no studies to date have explored the relationship among NAD^+^, cADPR, and ADPR with CIPN severity in peripheral blood among patients receiving neurotoxic chemotherapy. The primary purpose of this longitudinal, observational study was to determine the association between plasma NAD^+^ levels and CIPN severity in young adults receiving vincristine or paclitaxel. An exploratory aim was to determine the association between cADPR and ADPR with CIPN severity.

## Materials and methods

### Sample and setting

Young adults beginning vincristine or paclitaxel were recruited from the breast cancer, leukemia, and lymphoma disease centers at Dana-Farber Cancer Institute. Patients were eligible if they were 15–39 years old, English speaking, had a diagnosis of lymphoma, leukemia, or breast cancer, and planned to receive a cumulative vincristine dose of ≥ 7 mg or a cumulative paclitaxel dose of ≥ 700 mg/m^2^. Participants were excluded from study participation if they had a prognosis ≤ 3 months, neuropathy due to other causes (e.g., diabetes), planned to receive other neurotoxic agents (e.g., platinums) concurrently with vincristine and paclitaxel, were enrolled in symptom management trials that may alter CIPN severity, or previously received neurotoxic chemotherapy. While axon degeneration may be a common pathophysiological mechanism of CIPN among all neurotoxic agents, we only recruited young adults receiving vincristine or paclitaxel because both agents exert their antineoplastic effects via microtubule interference [7]. In addition, as there is mixed evidence surrounding whether young or old age predicts CIPN severity [20–22], by testing our aims in a young adult population, we attempted to decrease the possibility of participants’ age confounding the analyses. Study oversight was provided by the Dana-Farber/Harvard Cancer Center Office for Human Research Studies (19–862). Verbal consent was obtained from all study participants. A waiver of documentation of informed consent was approved by the institutional review board due to the minimal risk nature of the study and the need for social distancing due to the COVID-19 pandemic. Clinical trial number: not applicable.

### Measures

*European Organization of Research and Treatment of Cancer Quality of Life Questionnaire-Chemotherapy-Induced Peripheral Neuropathy Sensory and Motor Subscales (QLQ-CIPN20).* The QLQ-CIPN20 sensory (nine items) subscale measures participants’ self-reported severity of numbness, tingling, and pain in the hands/feet, while the motor subscale (eight items) measures participants’ self-reported loss of strength and/or associated functional limitations. Both subscales are scored from 0 to 100 (higher scores = worse CIPN) [23]. While there is evidence supporting the reliability and validity of the original subscales [24], recent data has called into question the stability of the subscale structure of the QLQ-CIPN20 and rather, provided support for scoring the measure as an additive checklist [25]. As such, numbness and tingling severity was also explored using the four items of the sensory subscale that ask about numbness or tingling in the hands and feet, respectively.

*NAD*^*+*^*and related metabolites.* Plasma was separated from whole blood according to standard operating procedure [26] by the Clinical Research Laboratory at Dana-Farber Cancer Institute following the participants’ blood draw. NAD^+^, cADPR, and ADPR were quantitatively profiled from plasma using a mass spectrometry-based metabolomics platform developed by the Dana-Farber Cancer Institute Metabolomics Core. Specifically, 1–3 ml of human plasma was extracted in 80% final volume methanol spiked with 13.5 nM isotopically labelled ^13^C_5_ NAD (Cambridge Isotope Laboratories, Inc). After extraction, samples were dried down in a centrifugal vacuum unit and reconstituted in 80% acetonitrile with 1% formic acid. Proteins and phospholipids were removed via Ostro pass-through plates (P/N 186005518, Waters). Of the resulting mixture, 20 ul of each sample was transferred to a glass vial for LC/MS analysis, and 5 ul of each sample was pooled together for a QC sample. Targeted measurements were conducted on a QExactive HF-X mass spectrometer equipped with a HESI II probe. The mass spectrometer was coupled to a Vanquish binary UPLC system (Thermo Fisher Scientific, San Jose, CA). For chromatographic separation prior to mass analysis, 5 ul of each metabolite extract was injected into an Atlantis Z-HILIC column (2.5 μm, 2.1 mm x 100 mm, Waters) equipped with a guard column (1.7 μm, 2.1 × 5 mm). Mobile phase B was 95% acetonitrile with 15 mM ammonium bicarbonate and Mobile phase A was 95% water with 15 mM ammonium bicarbonate. A gradient was applied as follows: 0–0.75 min, 95% B; 0.75–2.50 min, 77% B; 2.50–4.00 min, 77% B; 4.00–6.00, 50% B; 6.00–7.00 min, 50% B; 7.1 min, 95% B, 10.00 min, 95% B. Flow rate for chromatography was 500 µl min^− 1^. Full scan (*m/z* 70–900) negative mode data were acquired from 0 to 3.33 min and from 4.5 to 10 min; from 3.33 to 4.5 min, data were acquired in 4-plexed tSIM-mode with a 1.5 Da isolation window, 60,000 resolution, including the following ions: 558.06450, ADPR; 662.10250, NAD; 540.05380 cADPR; 667.11900, ^13^C_5_ NAD. The sheath gas flow was set to 40 units, the auxiliary gas flow to 8, and the sweep gas flow to 1 unit. Spray voltage was set to -3 kV. The injection order was randomized, a blank injection was conducted between each sample, and every 10 samples a QC block consisting of reference standards (purchased from Sigma; ADPR A0752; NAD 100-RO; NIST SRM 1950), and the pooled study sample was measured. A PRM experiment was performed on pooled study sample as well as authentic chemical standards for each analyte (N(CE) 25, 35, 45 V, resolution 30,000). For each sample, the retention time was determined based on retention time of the chemical standards in the preceding QC block. The target analytes were low abundance in many samples; therefore, an expert curation was performed to empirically establish a lower limit for the number of spectra in which a given analyte needed to be present. In most samples, seven or more scans over the chromatographic peak was deemed to result in good peak quality, and samples with fewer than seven scans were removed from subsequent analyses.

*Demographic and cancer treatment-related measures.* Participants self-reported demographic information such as age, gender, race, ethnicity, education, marital status, smoking status (i.e., current/never/former) [27, 28], and employment status. Alcohol use was measured using the three alcohol consumption items, modified for the United States standard drink size and guidelines [29, 30], of the Alcohol Use Disorders Identification Test (AUDIT) [31–33]. Each item is scored from 0 to 6 (0–18 total score; scores ≥ 7 for women and ≥ 8 for men indicate excessive drinking) [29, 30]. Self-reported anxiety and depression [34] were measured as potential confounders of CIPN development. The Patient-Reported Outcomes Measurement Information System (PROMIS^®^) Depression 4a measures participants’ perceptions of mood, views of self, and affect over the past week. Each item is scored from 1 to 5 (41.0–79.4 transformed total; higher scores = worse depression) [35–37]. The PROMISⓇ Anxiety 4a measures self-reported fearfulness, worry, nervousness, and uneasiness over the past week. Each item is scored from 1 to 5 (40.3–81.6 transformed total; higher scores = worse anxiety) [35, 37]. Study staff abstracted information about cancer type and stage, chemotherapy type and dose, medication use, and comorbid conditions from the participants’ electronic medical record.

### Procedures

Prior to the first paclitaxel or vincristine infusion, enrolled participants completed the QLQ-CIPN20, PROMIS Depression 4a, PROMIS Anxiety 4a, AUDIT, and the demographics questionnaire (T1). A blood sample was also obtained during the participants’ routine laboratory draw prior to chemotherapy at T1. The T2 and T3 follow up assessments were administered based on cumulative vincristine [38] (T2: 3–5 mg; T3: 7–9 mg) and paclitaxel [39] (T2: 350–450 mg/m^2^; T3: 700–900 mg/m^2^) dosages associated with increased CIPN incidence. At T2 and T3, participants completed the QLQ-CIPN20, PROMIS Depression 4a, PROMIS Anxiety 4a, and provided a blood sample. NAD^+^ and related metabolites were quantified from plasma at each time point.

### Statistical analysis

NAD^+^, cADPR, ADPR, and cADPR/NAD^+^ ratio levels and QLQ-CIPN20 scores (i.e., sensory subscale, motor subscale, and numbness and tingling items) at T2 and T3 were compared with T1 using a Wilcoxon signed-rank test. A generalized estimating equations (GEE) model accounting for repeated measures over time, and preliminarily assuming an independence correlation structure across time points was used to assess the association among NAD^+^ and QLQ-CIPN20 scores adjusting for anxiety and depression severity. Exchangeability between observations at specific time points was also tried as a sensitivity analysis, producing results very similar to the ones obtained assuming independence. The model outcomes were preliminarily transformed using a fourth root to approach as much as possible a normal distribution (tested using a Shapiro-Wilk test, *p*-value: 0.719). The analysis was stratified by chemotherapy type to determine changes in NAD^+^ and CIPN severity among young adults receiving paclitaxel or vincristine, respectively.

## Results

### Sample characteristics

**Fig. 1:**
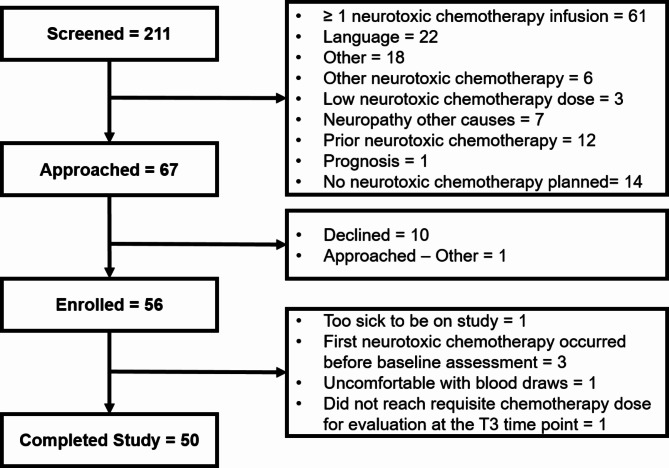
Participant flow diagram. This figure describes participants’ progress through the study

Figure 1 describes participant flow through the study. The primary reason for declining participation was related to being too busy to participate in research (e.g., overwhelmed with diagnosis or number of other appointments). Fifty-six participants were recruited from October 30, 2020 to September 7, 2022. Ultimately, 50 participants completed the study and were available for analysis. Table 1 describes the demographic and cancer treatment-related characteristics of the analyzed sample.

**Table 1:** Demographic characteristics of the enrolled sample at the time of consent (*N* = 50)

Characteristic	Frequency (%)
**Age at Consent**	
Emerging Adults (18–25 years old)	2 (4%)
Young Adult (26–39 years old)	48 (96%)
*Median* (*Range*)	35 (21–39)
**Sex**	
Female	44 (88%)
Male	6 (12%)
**Race**	
Asian	6 (12%)
Black or African-American	4 (8%)
White	40 (80%)
**Ethnicity**	
Hispanic or Latino	4 (8%)
Not Hispanic or Latino	43 (86%)
Missing	3 (6%)
**Education**	
Completed high school	4 (8%)
Some college or technical training	9 (18%)
University undergraduate degree	18 (36%)
University post graduate degree	19 (38%)
**Marital Status**	
Single	20 (40%)
Married/Partnered	28 (56%)
Divorced	1 (2%)
Missing	1 (2%)
**Employment Status**	
Working full time	22 (44%)
Working part-time	1 (2%)
Working at home	1 (2%)
Working, but on medical leave	16 (32%)
Not working	7 (14%)
Student	3 (6%)
**Smoking Status**	
Former Smoker	11 (22%)
Never Smoked	37 (74%)
Missing	2 (4%)
**Alcohol Use Disorders Identification Test**	
Positive^a^	5 (10%)
Negative	45 (90%)
*Median* (*Range*)	4 (0–13)
**PROMIS Anxiety 4a**	
T1 *Median* (*Range*)	59.8 (40.5–70.2)
T2 *Median* (*Range*)	52.9 (40.5–70.2) (*n* = 49)
T3 *Median* (*Range*)	54.5 (40.5–79.7)
**PROMIS Depression 4a**	
T1 *Median* (*Range*)	48.9 (41–71.1)
T2 *Median* (*Range*)	41 (41–74.4) (*n* = 49)
T3 *Median* (*Range*)	48.9 (41–79.3)
**Cancer Type**	
Leukemia	2 (4%)
Lymphoma	9 (18%)
Breast	39 (78%)
**Cancer Stage**	
Stage I	6 (12%)
Stage II	27 (54%)
Stage III	8 (16%)
Metastatic	6 (12%)
Unknown	3 (6%)
**Chemotherapy Type**	
Vincristine	11 (22%)
Paclitaxel	39 (78%)
**Cumulative Dose Paclitaxel (mg/m**^**2**^**)** (*n* = 39)	
T1 *Median (Range)*	0
T2 *Median (Range)*	350 (175–480)
T3 *Median (Range)*	700 (630–800)
**Cumulative Dose Vincristine (mg)** (*n* = 11)	
T1 *Median (Range)*	0
T2 *Median (Range)*	4 (2–6)
T3 *Median (Range)*	8 (6–10)
**Percentage of Planned Treatment at T3**	
Received < 1/3 of planned treatment	2 (4%)
Received ≥ 1/3 of planned treatment	1 (2%)
Received ≥ 2/3 of planned treatment	18 (36%)
Completed treatment	29 (58%)
**Days between T1 and T2**	
*Median* (*Range*) (*n* = 48)	28.5 (19–116)
**Days between T2 and T3**	
*Median* (*Range*) (*n* = 48)	33.5 (8–129)
**Days between T1 and T3**	
*Median* (*Range*)	72 (27–157)
**Days between end of treatment and T3**	
*Median* (*Range*) (*n* = 29)	35 (4–101)
**Baseline Medications** ^b^	
Yes	2 (4%)^c^
None	48 (96%)
**T3 Medications** ^b^	
Yes	8 (16%)^d^
None	42 (84%)
**Neurotoxic Chemotherapy Dose Reduction**	
Yes, neuropathy-related	2 (4%)
Yes, other reasons^e^	7 (14%)
No	41 (82%)

Notes

^a^ Scores ≥ 7 and 8 indicate a positive score for women and men, respectively (e.g., at risk of hazardous drinking behaviors)

^b^ Represent medications that were documented in participants’ medical records at a given time point that could potentially influence CIPN severity

^c^ One participant was receiving gabapentin and cryotherapy, while another was receiving gabapentin

^d^ Seven participants were receiving gabapentin, one participant was receiving vitamin B complex

^e^ Other reasons for neurotoxic chemotherapy dose reduction or delay included rash, diarrhea, neutropenia, elevated liver function testes, weight loss/failure to thrive

### Association between metabolite levels and patient-reported CIPN

Table 2 describes changes in CIPN patient-reported outcomes scores and metabolite levels as the cumulative chemotherapy dose increased from T1 to T3. Overall, QLQ-CIPN20 sensory, motor, and numbness and tingling (*p* < 0.001) severity scores significantly increased from T1 to T3. NAD^+^ (*p* = 0.28), cADPR (*p* = 0.62), and ADPR (*p* = 0.005) values decreased from T1 to T3, while the cADPR/NAD^+^ ratio increased from T1 to T3 (*p* = 0.50). There was no statistically significant association between NAD + and QLQ-CIPN20 sensory score over time (*p* = 0.28). Similar relationships were observed among changes in QLQ-CIPN20 motor and numbness and tingling item scores with NAD^+^, respectively (Table 3).

**Table 2:** Patient-reported outcome scores and metabolite levels from T1 to T3 (*N* = 50)

Measure	T1	T2	T3	T1 – T2 Change	T1 – T3 Change
**QLQ-CIPN20 Sensory**	2.22 (7.7)	9.15 (13.44)	7.41 (10.26)	6.88 (12.87)*	5.19 (10.23)*
**QLQ-CIPN20 Motor**	2.35 (8.17)	9.06 (18.24)	6.95 (11.26)	6.67 (14.22)*	4.61 (8.47)*
**QLQ-CIPN20 Numbness and Tingling**	3.17 (10.36)	14.12 (19.19)	13.17 (16.76)	10.88 (18.18)*	10.0 (16.92)*
**NAD** ^**+ a**^	1.34e^− 07^ (9.22e^− 08^)	1.28e^− 07^ (1.02e^− 07^)	1.2e^− 07^ (1.23e^− 07^)	1.08e^− 8^ (1.49e^− 07^)	-1.4e^− 08^ (1.24e^− 07^)
**cADPR** ^**b**^	6.65e^− 08^ (6.96e^− 08^)	6.10e^− 08^ (5.17e^− 08^)	6.23e^− 08^ (7.34e^− 08^)	-3.8e^− 09^ (8.47e^− 08^)	-4.2e^− 09^ (7.68e^− 08^)
**ADPR** ^**c**^	2.44e^− 07^ (6.43e^− 07^)	7.98e^− 08^ (9.26e^− 08^)	1.83e^− 07^ (5.17e^− 07^)	-1.64e^− 07^ (-6.26e^− 07^)	-1.93e^− 07^ (9.95e^− 07^)*
**cADPR/NAD** ^**+ d**^	0.63 (0.33)	0.79 (0.50)	0.71 (0.40)	0.13 (0.60)	0.08 (0.39)

Notes:

Table 2 describes *mean* (*SD*) metabolite and patient-reported CIPN scores at T1, T2, and T3. NAD^+^, cADPR, ADPR, and cADPR/NAD^+^ ratio levels and QLQ-CIPN20 scores at T2 and T3 were compared with T1 using a Wilcoxon signed-rank test. Relative abundance as normalized mass spectrometer signal intensity is shown for NAD^+^, cADPR, and ADPR

*p < 0.05

^a^ T1 & T3 *n* = 25, T2 *n* = 20

^b^ T1 & T3 *n* = 35, T2 *n* = 32

^c^ T1 & T3 *n* = 31, T2 *n* = 29

^d^ T1 & T3 *n* = 23, T2 *n* = 19

**Table 3:** Association among NAD^+^ levels and QLQ-CIPN20 subscales scores over Time

Variable	Whole Sample		Paclitaxel		Vincristine	
	Estimate	*p*	Estimate	*p*	Estimate	*p*
**QLQ-CIPN20 Sensory Subscale**	-0.02	0.28	-0.02	0.41	-0.05	0.61
**Anxiety**	-0.02	0.60	0.009	0.84	-0.08	0.24
**Depression**	-0.05	0.27	-0.08	0.12	-0.01	0.96
**Time**	0.09	0.79	-0.02	0.96	0.63	0.54
	**Estimate**	***p***	**Estimate**	***p***	**Estimate**	***p***
**QLQ-CIPN20 Motor Subscale**	-0.02	0.17	-0.01	0.57	-0.07	0.08
**Anxiety**	-0.02	0.54	0.01	0.82	-0.08	0.23
**Depression**	-0.04	0.30	-0.08	0.13	-0.001	0.99
**Time**	0.09	0.79	-0.02	0.95	0.57	0.52
	**Estimate**	***p***	**Estimate**	***p***	**Estimate**	***p***
**QLQ-CIPN20 Numbness and Tingling Items**	-0.02	0.22	-0.02	0.15	-0.01	0.81
**Anxiety**	-0.02	0.58	0.003	0.94	-0.08	0.29
**Depression**	-0.05	0.26	-0.07	0.16	-0.003	0.98
**Time**	0.12	0.71	0.01	0.97	0.60	0.58

Notes:

A GEE model accounting for repeated measures across the three time points (time), and preliminarily assuming an independence correlation structure across time points was used to assess the association among the 4th root of the NAD^+^ area and QLQ-CIPN20 scores adjusting for anxiety and depression severity. The values approximate the change in the 4th root of the NAD^+^ area associated with a one-point increase in the respective variables

Among young adults treated with paclitaxel, NAD^+^ (*mean* = -7.1e^− 09^, *p* = 0.42, *n* = 22), cADPR (*mean* = -1.4e^− 09^, *p* = 0.94, *n* = 29), and ADPR (*mean* = -2.42e^− 07^, *p* = 0.02, *n* = 22) decreased from T1 to T3, while the cADPR/NAD^+^ ratio increased (*mean* = 0.1, *p* = 0.35, *n* = 20) from T1 to T3. cADPR/NAD^+^ ratio increased by 0.21 from T1 to T2 (*p* = 0.19, *n* = 15) (Supplementary Table 1). Results of the GEE models indicated that for each one-point increase in QLQ-CIPN20 sensory scores, NAD^+^ decreased by 0.02 (*p* = 0.41). Similar relationships were observed among changes in QLQ-CIPN20 motor and numbness and tingling item scores with NAD^+^, respectively (Table 3).

Among young adults treated with vincristine, NAD^+^ (*mean* = -6.43e^− 08^, *p* = 0.50, *n* = 3) cADPR (*mean* = -1.79e^− 08^, *p* = 0.31, *n* = 6), ADPR (*mean* = -3.57e^− 08^, *p* = 0.22, *n* = 7) and the cADPR/NAD^+^ ratio (*mean* = -0.11, *p* = 0.75, *n* = 3) decreased from T1 to T3 (Supplementary Table 2). Results of the GEE models indicated that for each one-point increase in QLQ-CIPN20 sensory scores, NAD^+^ decreased by 0.05 (*p* = 0.61). Similar relationships were observed among changes in QLQ-CIPN20 motor and numbness and tingling item scores with NAD^+^, respectively (Table 3).

## Discussion

To our knowledge, this is among the first human studies to measure longitudinal changes in plasma NAD^+^, cADPR, and ADPR as markers of axon degeneration among patients receiving neurotoxic chemotherapy. Inconsistent with prior preclinical studies [10, 11, 15, 16], results of the GEE models revealed non-significant decreases in NAD^+^ levels as CIPN severity worsened over time. A difference between prior preclinical work and the present study is that the preclinical work demonstrated changes in NAD^+^ following axon injury at one time point [11, 15, 40], whereas the present study explored changes in NAD^+^ over time. It is plausible that plasma NAD^+^ levels were influenced by other variables not controlled for in the analysis (e.g., diet, exercise, sleep habits, aging [41], other NAD^+^ cleaving enzymes) [19]. For such reasons, NAD^+^ is currently not considered to be the best biomarker of SARM1 activity [18].

cADPR and ADPR levels also decreased from T1 to T3 along with NAD^+^, which is inconsistent with prior studies demonstrating that SARM1-induced breakdown of NAD^+^ leads to the increased generation of nicotinamide, cADPR, and ADPR [15]. SARM1 activity generates cADPR and Li et al. (2022) demonstrated that increased cADPR production following SARM1 activity promoted intra-axonal calcium influx that precedes paclitaxel-induced axonal degeneration [17]. ADPR may not be considered the most promising candidate biomarker of SARM1 activity as ADPR is largely produced by CD38 [42] and can be quickly metabolized in the cell following increased SARM1-induced ADPR generation [19], which may be a rationale for why we observed decreases in ADPR. Nonetheless, despite the conflicting patterns in NAD^+^, cADPR, and ADPR changes over time, we observed non-significant increases in the ratio of cADPR/NAD^+^, particularly between T1 and T2 among patients receiving paclitaxel. Thus, while preliminary, the changes observed in the ratio of cADPR/NAD^+^ over time provide evidence supporting increased SARM1 activity among patients receiving paclitaxel.

Further research to explore the ratio of cADPR/NAD^+^ as a biomarker of axon degeneration related to CIPN may be worthwhile and would provide complimentary data to the information provided by neurofilament light chain levels (i.e., structural components of axons) [43]. The measurement of cADPR and the cADPR/NAD^+^ ratio are currently thought to measure subdegenerative levels of SARM1 activity and may be useful in mechanistic studies [19], while neurofilament light chain levels are thought to measure axonal loss [19]. Several studies have explored neurofilament light chain levels in peripheral blood as a biomarker of axonal damage among patients receiving paclitaxel (*N* = 349 across approximately five studies) [43]. Study results have generally shown that neurofilament light chain levels increase as cumulative paclitaxel dose increases and changes in neurofilament light chain levels are associated with worsening CIPN [43]. Clinically, the biomarkers are not routinely used in practice for CIPN monitoring and treatment decision making [44]. The potential validation of the ratio of cADPR/NAD^+^ and neurofilament light chains as biomarkers of CIPN and the timing of such changes will provide clinicians with mechanistic data to corroborate patients’ report of CIPN and to potentially guide decisions as to when to dose reduce chemotherapy or initiate other treatments for CIPN.

### Limitations

There are several limitations to this research. CIPN severity was measured using patient-reported outcomes only and the timing of CIPN measures may have been suboptimal given that 58% of participants completed neurotoxic chemotherapy by the T3 time point. The high number of participants that completed neurotoxic chemotherapy by T3 may explain why a decrease in CIPN severity occurred at T3 in comparison to T2 (i.e., CIPN has been demonstrated to decrease after neurotoxic chemotherapy completion) [45]. We may have observed more severe CIPN among the sample if patient-reported CIPN severity was measured at the last paclitaxel or vincristine infusion for each individual instead of at specific cumulative dosages. We were unable to initiate recruitment from the pediatric oncology clinic at Dana-Farber Cancer Institute during the COVID-19 pandemic and thus, no adolescents (< 18 years old) were enrolled. Plasma was extracted from whole blood approximately one hour after the blood draw. It is unclear if this is the best timing for plasma extraction from whole blood to optimally measure the metabolites of interest or if we would have seen different changes if the plasma was extracted from whole blood immediately after the blood draw. Similarly, given that blood was drawn prior to neurotoxic chemotherapy administration, we were unable to capture immediate changes in metabolite levels in response to neurotoxic chemotherapy exposure (e.g., does NAD^+^ decrease in response to NMNAT2 loss and/or as a result of increased SARM1 activity) [11, 14]. While the results suggest a trend in cADPR/NAD^+^ ratio from T1 to T2, simulations based on our pilot data reveal that we would need cADPR and NAD^+^ metabolite data from 174 participants at each respective time point to have adequate power to detect a ~ 25% difference in mean change of cADPR/NAD^+^ ratio over the same time points during neurotoxic chemotherapy in a future study. Finally, while we attempted to control for confounding variables in our eligibility criteria, several other factors (e.g., lifestyle, nutrition, physical activity, or genetics) [46] that were not measured may have influenced CIPN severity and hindered the ability to determine the relationship between CIPN severity and the axon degeneration-related biomarkers of interest in this small sample.

## Conclusions

Overall, study results demonstrated that plasma NAD^+^ and cADPR did not significantly change over time among young adults receiving paclitaxel or vincristine. The results also suggested that cADPR/NAD^+^ ratio increased over time, a potential biomarker of SARM1 activity, but the changes were not statistically significant and the increases were mainly observed among patients receiving paclitaxel. The study results are revealing, as this is the first study to measure plasma NAD^+^, cADPR, and ADPR among patients receiving neurotoxic chemotherapy. Future research is needed to validate and explore the clinical utility of biomarkers of axon degeneration, such as cADPR/NAD^+^ ratio, among patients receiving neurotoxic chemotherapy to guide mechanistic research and novel treatments for CIPN.

## Electronic supplementary material

Below is the link to the electronic supplementary material.

Supplementary Material 1

## Data Availability

Raw instrument data and experiment metadata associated with the mass spectrometry experiments will be uploaded to Metabolights (https://www.ebi.ac.uk/metabolights). The clinical and patient-reported outcomes data used and/or analyzed during the current study are available from the corresponding author on reasonable request.

## Abbreviations

ADPR: Adenosine diphosphate ribose
AUDIT: Alcohol Use Disorders Identification Test
cADPR: Cyclic adenosine diphosphate ribose
CIPN: Chemotherapy–induced peripheral neuropathy
GEE: Generalized estimating equations
NAD^+^: Nicotinamide adenine dinucleotide
NMNAT2: Nicotinamide mononucleotide adenylyltransferase 2
NMN: Nicotinamide mononucleotide
PROMIS: Patient–Reported Outcomes Measurement Information System
QLQ-CIPN20: European Organization of Research and Treatment of Cancer Quality of Life Questionnaire-Chemotherapy-Induced Peripheral Neuropathy Sensory and Motor Subscales
SARM1: Sterile alpha and Toll/interleukin 1 receptor motif containing protein 1
