# Supplementary material for: Exploring clinical markers of Axon degeneration processes in Chemotherapy-induced peripheral neuropathy among young adults receiving vincristine or paclitaxel
Source: BMC Neurol. 2024 Sep 28;24:366. doi: 10.1186/s12883-024-03877-9 (PMC11438373; doi:10.1186/s12883-024-03877-9)
Supplement: Supplementary file 1 — Supplementary Material 1 [file 12883_2024_3877_MOESM1_ESM.docx]

**Supplementary Table 1**

**Patient-Reported Outcome Scores and Metabolite Levels from T1 to T3 Among Participants Receiving Paclitaxel (*N* = 39)**

| **Measure** | **T1** | **T2** | **T3** | **T1 – T2 Change** | **T1 – T3 Change** |
| --- | --- | --- | --- | --- | --- |
| **QLQ-CIPN20 Sensory** | 2.56 (8.63) | 9.84 (14.34) | 7.22 (10.51) | 7.21* (13.61) | 4.65*  (10.33) |
| **QLQ-CIPN20 Motor** | 2.05 (8.76) | 8.62 (19.27) | 1.17  (0.34) | 6.52* (15.29) | -0.87* (8.50) |
| **QLQ-CIPN20 Numbness and Tingling** | 3.85 (11.61) | 15.13 (20.03) | 12.39 (16.43) | 11.18* (18.71) | 8.55* (16.39) |
| **NAD^+a^** | 1.36e^-07^ (9.42e^-08^) | 1.06e^-07^ (7.75e^-08^) | 1.29e^-07^ (1.28e^-07^) | -1.52e^-8^ (1.25e^-07^) | -7.1e^-09^ (1.29e^-07^) |
| **cADPR^b^** | 7.10e^-08^ (7.41e^-08^) | 6.46e^-08^ (5.45e^-08^) | 6.97e^-08^ (7.87e^-08^) | -5.9e^-09^ (9.44e^-08^) | -1.4e^-09^ (8.32e^-08^) |
| **ADPR^c^** | 2.81e^-07^ (7.12e^-07^) | 7.19e^-08^ (8.88e^-08^) | 2.27e^-07^ (5.89e^-07^) | -2.09e^-07^  (6.88e^-07^) | -2.42e^-07^* (1.14e^-06^) |
| **cADPR/NAD^+d^** | 0.63  (0.35) | 0.87  (0.50) | 0.73  (0.41) | 0.21  (0.60) | 0.10  (0.38) |

**Notes:**

Supplementary Table 1 describes *mean* (*SD*) metabolite and patient-reported CIPN scores at T1, T2, and T3 among patients receiving paclitaxel. NAD^+^, cADPR, ADPR, and cADPR/NAD^+^ ratio levels and QLQ-CIPN20 scores at T2 and T3 were compared with T1 using a Wilcoxon signed-rank test. Relative abundance as normalized mass spectrometer signal intensity is shown for NAD^+^, cADPR, and ADPR. The footnotes refer to the available sample size at each time point for the selected outcomes.

***** *p <0.05*

^a^ T1 & T3 *n* = 22, T2 *n* = 16

^b^ T1 & T3 *n* = 29, T2 *n* = 25

^c^ T1 & T2 *n* = 25, T3 *n* = 22

^d^ T1 & T3 *n* = 20, T2 *n* = 15

**Patient-Reported Outcome Scores and Metabolite Levels from T1 to T3 Among Participants Receiving Vincristine (*N* = 11)**

| **Measure** | **T1** | **T2** | **T3** | **T1 – T2 Change** | **T1 – T3 Change** |
| --- | --- | --- | --- | --- | --- |
| **QLQ-CIPN20 Sensory** | 1.01 (2.40) | 6.73* (9.91) | 8.08  (9.77) | 5.72 (10.39) | 7.07* (10.14) |
| **QLQ-CIPN20 Motor** | 3.41 (5.84) | 10.61 (14.80) | 1.33  (0.33) | 7.20* (10.25) | -2.08  (5.83) |
| **QLQ-CIPN20 Numbness and Tingling** | 0.76 (2.51) | 10.61 (16.28) | 15.91 (18.43) | 9.85  (17.0) | 15.15* (18.57) |
| **NAD^+a^** | 1.02e^-07^ (8.06e^-08^) | 2.17e^-07^ (1.5e^-07^) | 5.21e^-08^ (4.14e^-08^) | 1.15e^-7^ (2.13e^-07^) | -6.43e^-08^ (6.84e^-08^) |
| **cADPR^b^** | 4.47e^-08^ (3.49e^-08^) | 4.84e^-08^ (4.07e^-08^) | 2.67e^-08^ (1.37e^-08^) | 3.7e^-09^ (3.72e^-08^) | -1.79e^-08^ (3.29e^-08^) |
| **ADPR^c^** | 8.01e^-08^ (1.23e^-07^) | 1.13e^-07^ (1.09e^-07^) | 4.44e^-08^ (5.89e^-08^) | 2.5e^-08^  (1.62e^-07^) | -3.57e^-08^ (6.73.e^-08^) |
| **cADPR/NAD^+d^** | 0.64 (0.18) | 0.49  (0.45) | 0.55  (0.28) | -0.16  (0.58) | -0.11  (0.46) |

**Notes:**

Supplementary Table 2 describes *mean* (*SD*) metabolite and patient-reported CIPN scores at T1, T2, and T3 among patients receiving vincristine. NAD^+^, cADPR, ADPR, and cADPR/NAD^+^ ratio levels and QLQ-CIPN20 scores at T2 and T3 were compared with T1 using a Wilcoxon signed-rank test. Relative abundance as normalized mass spectrometer signal intensity is shown for NAD^+^, cADPR, and ADPR. The footnotes refer to the available sample size at each time point for the selected outcomes.

***** *p <0.05*

^a^ T1 & T2 *n* = 4, T3 *n* = 3

^b^ T1 & T2 *n* = 7, T3 *n* = 6

^c^ T1 & T3 *n* = 7, T2 *n* = 6

^d^ T1 & T2 *n* = 4, T2 *n* = 3
